# Assessment of root resorption on lateral incisors after primary canine extraction treating mesioangular displaced permanent canines: a randomised controlled trial

**DOI:** 10.3389/fdmed.2024.1456985

**Published:** 2025-01-21

**Authors:** Narmin Helal, Reem Naaman, Najlaa Alamoudi, Azza El-Housseiny, Fatima Jadu

**Affiliations:** ^1^ Pediatric Dentistry Department, King Abdulaziz University, Jeddah, Saudi Arabia; ^2^ Pediatric Dentistry Department, Taibah University, Medina, Saudi Arabia

**Keywords:** disturbances in dental development, growth and development, occlusion/orthodontics, radiology, canine impaction, root resorption (RR)

## Abstract

**Aim:**

Root resorption (RR) of the adjacent maxillary lateral incisors is considered the most common complication of displaced maxillary canines. The aim of this study was to assess the effect of interceptive extraction of the primary canines on the condition of the roots of permanent neighbouring teeth to mesioangular displaced canines (MDC). In addition, this study aimed to investigate the relationship between the position of the unerupted canine and the risk of RR in adjacent lateral incisors.

**Design:**

Randomised controlled clinical trial.

**Methods:**

Eighty-five patients 9–12-year-old with MDC were allocated equally to either an extraction group (EG) or a control group (CG). Of the sample, 33 were males (38.8%), and 52 were females (61.2%). Among this sample, 81 canines (51.9%) were buccally displaced, 34 canines (21.8%) were palatally displaced, and 41 canines (26.3%) were mid-alveolar. All subjects underwent cone-beam computed tomography examination to determine the presence, stage, and level of lateral incisor root resorption (RR). Measurements were performed at T0 and repeated at a 12-month follow-up (T2).

**Results & statistics:**

Root resorption (RR) was present in 28.2% of lateral incisors at the baseline assessment (T0) and exhibited a significant increase at the 12-month follow-up (T12). This marked increase in resorption severity was evident between T0 and T12 (*P* = 0007), regardless of group allocation. There was a significant increase in the degree of resorption between T2 and T0, with a mean difference equals to 0.31 (0.73), *P* < 0.0001. While there was no statistically significant difference in resorption levels between the extraction and control groups at T0 (*P* = 0.11), RR occurred more frequently with midalveolar (34.1%) and palatal (32.3%) displacements than with buccal displacements (23.5%). Severe resorption was observed more frequently in cases involving palatally displaced canines within both groups, but it did not reach statistical significance.

**Conclusions:**

Lateral incisor root resorption (RR) progressed significantly within a 12-month period irrespective of treatment modality. RR occurred more frequently with midalveolar and palatal displacement with a trend towards increased RR severity in cases with palatally displaced canines. Early clinical evaluation and consistent monitoring is essential for early detection and potential intervention in patients with mesioangular canine displacement.

## Introduction

Untreated canine displacement can cause several complications, such as external root resorption (RR) of the canine and adjacent teeth, follicular cyst formation, recurrent pain and infection, canine ankylosis, migration of neighbouring teeth, and shortening of the arch length space (1, 2). RR of the adjacent maxillary lateral incisors is considered the most common complication of displaced maxillary canines. It is defined as the loss of root cementum and/or dentin, which is the result of the physiological or pathological activity of the osteoclasts (1). Many studies have reported that palatally displaced canines (PDCs) can cause RR of adjacent incisors' roots; nevertheless, resorption can also be caused by buccally displaced canines (3). The process of RR is asymptomatic and is usually diagnosed late, at a more advanced stage when it becomes difficult to treat (4). Severe resorption can be detected as early as 9 years of age (5). Two-dimensional (2D) radiographs have limitations in diagnosing canine displacement related RR. They lack the ability to provide information regarding the third dimension, namely, the labiopalatal dimension, which is important in cases of impacted maxillary canines (6). Therefore, it is not possible, for example, to detect mild resorption located in the buccal or palatal surfaces of incisors or canine roots (1, 7). Three-dimensional (3D) imaging has been shown to be superior to 2D imaging in the localisation of impacted canines and in evaluating the degree of RR (8, 9). Haney et al. reported a 36% difference in RR between 3D and 2D, which is in line with the findings of a classical study by Ericson and Kurol in 1987 (10) where they mentioned that only 50% of the resorptions were seen when using periapical radiographs only. This was also confirmed by a recent study, but more importantly, 30% of the severe resorption cases detected by computed tomography (CT) imaging were completely missed by intraoral films (5). Another study showed that resorption of lateral incisors was detected more often with CBCT than with panoramic radiographs, 18% and 11.5%, respectively (11). Compared to the visual inspection of the extracted lateral incisor roots, CT scans were found to be in a high level of agreement. Ericson and Kurol have also contributed to modifications in the treatment plan for children with displaced canines (12). In 2006, Bjerklin and Ericson reported that more than 53% of the original treatment plans for children with PDCs and incisor RR were altered after CT investigation (13).

Diagnosis and treatment planning of impacted teeth and assessment of RR are considered a justified indication for the use of cone-beam CT (CBCT) in children according to the DIMITRA (Dentomaxillofacial paediatric imaging: an investigation toward low-dose radiation induced risk) project (14).

The aetiology of RR is not well understood and remains unclear. However, direct contact between the displaced canine and lateral incisor increases the risk of RR (1, 3, 5). Canines can cause resorption most commonly to the roots of lateral incisors, but can also affect the roots of the central incisors and maxillary premolars (1, 15, 16). The underlying mechanisms contributing to root resorption in such scenarios are complex and multifaceted. The new theory of eruption suggests that the process involves three key structures: the periodontal membrane, the crown follicle, and the tooth's apical region. Pressure in the apical region stimulates the crown follicle to resorb surrounding bone tissue, while the periodontal membrane adapts to facilitate eruption. Pathological course can take place in any of this discussed process. For example, Trauma or other disturbances can lead to inflammation of the periodontal membrane, which can result in fluid accumulation and subsequent tissue damage (17). Previous research has explored various factors that may influence root resorption, including the size, shape, and position of the dental follicle associated with the impacted canine (18). However, the specific role of the dental follicle's volume in tooth displacement remains a subject of debate. Some studies suggest that the hydraulic pressure exerted by the dental follicle's contents can contribute to root resorption, while others emphasize the eruptive force of the canine itself. There was no significant correlation was found between dental follicle volume and the mesiodistal tip, buccolingual torque, or mesiodistal rotation of the adjacent lateral incisors and first premolars (19). The eruptive process or migration of the unerupted canine during root growth can increase the risk of RR. Physical proximity between the unerupted canine and neighboring roots, particularly when less than 1 mm, is a significant predictor of RR. The resorption of neighboring roots is likely triggered by a combination of factors, including direct physical injury, increased force on the local root cementum, and the release of resorptive molecules from the canine follicle as mentioned above. Understanding these mechanisms is crucial for developing effective prevention and treatment strategies (20). Given the lack of consensus on these mechanisms and the scarcity in the literature regarding the assessment of RR neighbouring mesially displaced canines with follow-up, there is a pressing need for clinical evidence to guide prevention and treatment strategies for RR in cases of canine displacement. In this context, the current study aims to evaluate the impact of early primary canine extraction on the condition of the permanent lateral incisor roots, with a focus on RR, in patients with mesioangular canine displacement (MDC). Additionally, we aim to examine the relationship between the severity of lateral incisor RR and specific patterns of canine displacement. By addressing these objectives, this study seeks to enhance our understanding of RR in canine displacement and to contribute evidence-based recommendations for clinical decision-making.

## Materials and methods

This study was approved by the Research Ethics Committee of the Faculty of Dentistry (REC-FD) on 20 September 2018 (Proposal No. 073-09-17) and registered at clinicaltrial.gov (NCT03684525). Procedures followed were in accordance with the ethical standard of the Helsinki Declaration of 1975. Participants were recruited at King Abdulaziz University Dental Hospital (UDH) between March 2017 and February 2019. Written informed consent was obtained from the legal guardians of each patient. The inclusion and exclusion criteria were as follows.

### Inclusion criteria

(1) Children's age at diagnosis had to be between 9 and 12 years old.
(2) Unilateral or bilateral mesioangular displaced maxillary canines had to be identified using panoramic radiographs taken 3 to 6 months before the day of evaluation and of good diagnostic quality.
(3) Clinically, the maxillary primary canines should be present.

### Exclusion criteria

Participants who did not meet the inclusion criteria were excluded from the study.

(1) Any child with systemic condition, craniofacial syndromes or cleft lip and/or palate.
(2) Clinically, any child with previous, ongoing, or who had started orthodontic treatment at any point in the study.
(3) Children with severe arch length discrepancy, anterior and posterior crossbites.
4. Children with bilateral congenitally missing maxillary lateral incisors.

### Imaging exclusion criteria

(1) Poor-quality panoramic radiographs, related to patient positioning or to exposure parameters.
(2) Maxillary canines with severe displacement at diagnosis; Any type of canine displacement other than mesioangular displacement; Trans-positioned canines or closed apices
(3) Presence of pathology surrounding the canine (such as a cyst, supernumerary, and odontome).
(4) Early stages of canine root development (stages 0–6) were described by Nolla (21).

### Sample size and power calculation

An analysis was carried out on the full analysis set, including the maximum possible number of randomised participants. The sample size was calculated based on the results of Naoumova et al. (22, 23), who reported the successful eruption of permanent canines during the total follow-up period (primary outcome) for a 30% difference in palatally displaced canines' (PDCs) eruption rates between the extraction group (69%) and non-extraction group (39%). Using *α* level of 0.05 and *β* level of 0.20 (80% power), a 30% difference was reported between the groups. A two-sided Pearson's *χ*^2^ test with normal approximation indicated that a total of 86 patients were needed, i.e., 43 patients in each group.

### Study design

This randomised controlled clinical trial followed the Consolidated Standards of Reporting Trials (CONSORT) guidelines (24). A sample of 86 patients was allocated equally to one of the two groups as follows: Test group (EG; extraction of primary maxillary canines) = 43 patients and Control group (CG; no extraction of primary maxillary canines) = 43 patients.

### Randomisation, allocation, and blinding

Panoramic radiographs were evaluated by two calibrated examiners to diagnose canine displacement based on factors including canine overlap, inclination (25), and vertical position (26). All eligible screened cases had permanent canines that overlapped lateral incisors on sector 2 or 3, with an angular inclination of more than 15 degrees (25) and a vertical height grade of 2 or 3 (26). All participants who were eligible to participate in the trial were randomised into one of the two study groups, namely, the test group (EG) or the control (CG). Patients are matched in each group based on age, level of canine displacement and stage of root development. A randomised block design (27) was created by the biostatistician. The block size was 4 or 6; the lengths were randomly chosen, with equal probability, and the length of any given block was unknown to investigators and clinic personnel. This block design assured a balanced allocation in the two groups. In addition, it reduced the ability for the investigator to guess the next treatment group's assignment, thus minimising any unconscious bias in patient allocation into the different treatment groups. Patient allocation was concealed and numbered sequentially. A sealed opaque envelope containing the group assignment was given to the clinical personnel after written consent had been obtained, and patients were assigned randomly to either group.

Baseline CBCT image measurements were double-blinded. The principal investigator was unaware of the group the patients had been allocated. However, the 12-month follow-up images were not measured blindly because it was not possible to hide the extraction site. Nonetheless, at the time of measurements of the follow-up images, the examiner was unaware of the results of the previous image. Thus, the investigator was unable to compare the readings.

### Calibration of examiners

Several calibration tests were performed for panoramic radiographic evaluation by two examiners: an orthodontist and a paediatric dentist. This was done to assess the intra-and inter-examiner reliability tests.

In the evaluation of panoramic radiographs, intra-examiner reliability obtained by the first and second examiner was 0.87 and 0.88, respectively, demonstrating an almost perfect intra-rater agreement. The inter-rater reliability was 0.63, indicating a substantial inter-rater agreement. In the evaluation of CBCT scans, the intra-class correlation coefficient (ICC) was used to assess the intra-examiner reliability, which was good on average (ICC = 0.8786) for all the following variables: mesioangular angle, canine-to-lateral angle, sagittal angle, vertical position, cusp tip to midline, cusp tip to dental arch, and tooth resorption. Tooth resorption scored 0.830 on the ICC.

### CBCT assessment at baseline (T0)

**Figure 1:**
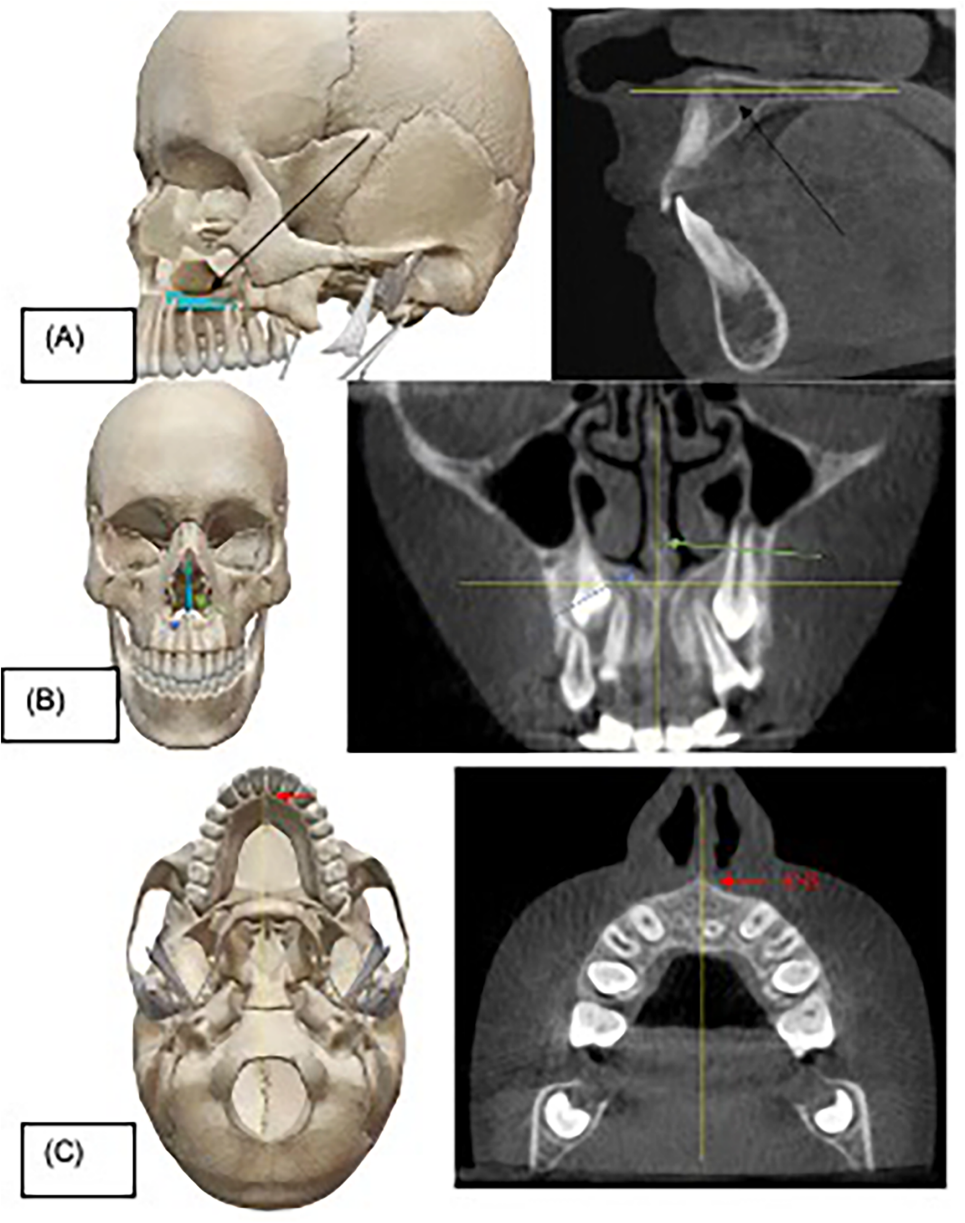
Head position adjustment before taking measurements. **(A)** Dry skull showing the palatine process of maxilla (black arrow) that was used as a reference in sagittal view of cone-beam computed tomography (CBCT) image. **(B)** Dry skull showing the nasal septum (green arrow) and nasal floor (blue arrow) which were used as references in coronal view of CBCT image. **(C)** Dry skull showing the anterior nasal spine (ANS) (red arrow) used to help adjust the midsagittal plane to be perpendicular to the ground. Yellow lines reflect the correct head position in all three planes.

**Figure 2:**
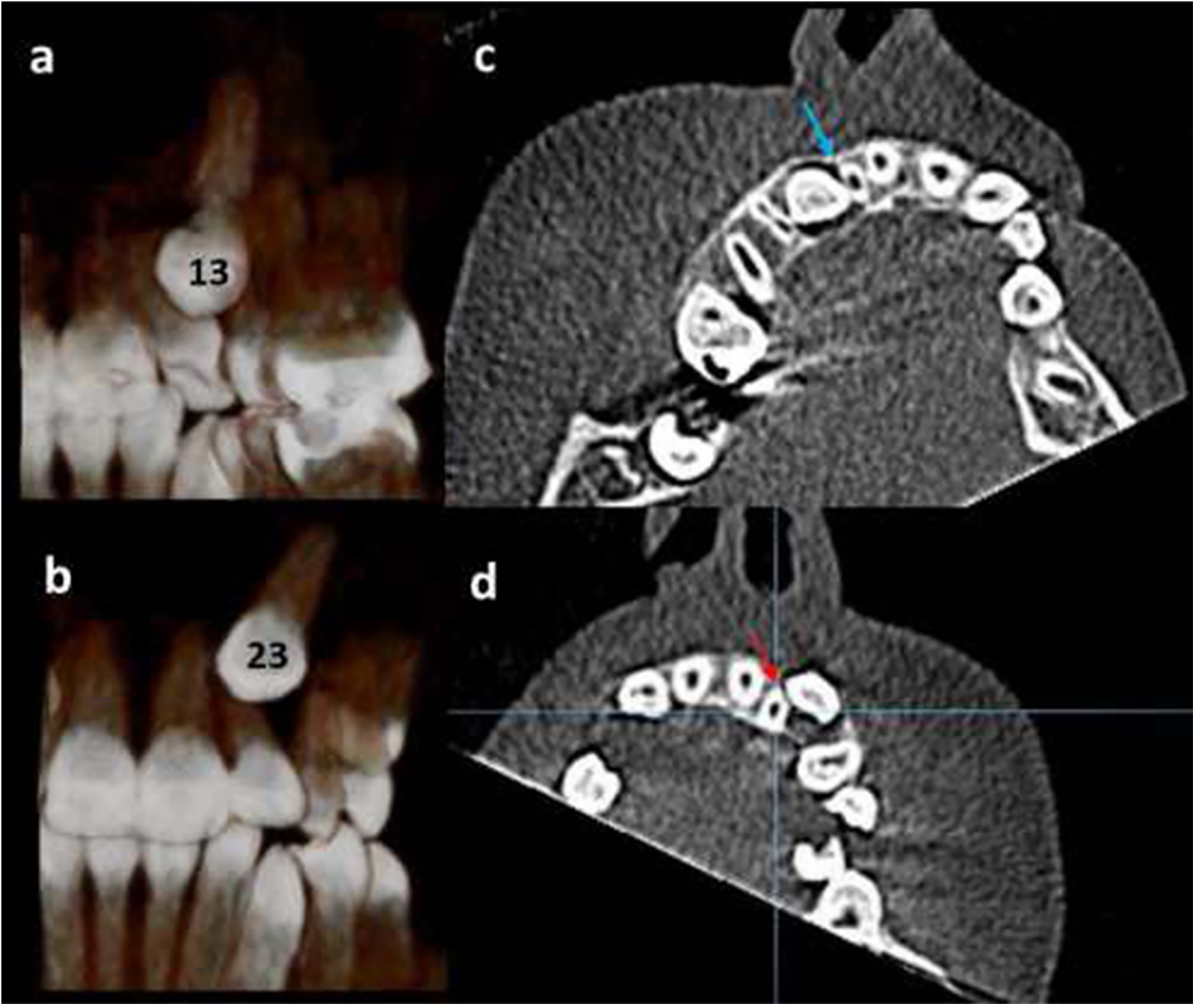
12-year-old girl, with bilateral displaced maxillary canines; **(a)** right canine “13” is in palatal position (three-dimensional palatal view). **(b)** left canine “23” is in buccal position (three-dimensional frontal view). **(c)** The right maxillary lateral incisor has Grade 3 resorption (blue arrow). **(d)** The left maxillary lateral incisor has Grade 2 root resorption (red arrow).

After collection of all the required information, all subjects underwent a preoperative CBCT examination to take measurements of the canine position, and to assess any presence of RR of maxillary lateral incisors using the I-CAT® (KaVo, USA) Imaging System with the following settings: field of view (FOV) size: 16 × 6 cm (small), resolution (Voxel size): 0.4 voxel, scan time: 4.8 s (Quick-Scan protocol); all participants used lead apron and thyroid collar. The head position of the patient was adjusted in all three planes (axial, coronal, and sagittal). Sagittally, the palatine process of the maxilla was parallel to the ground. The midsagittal plane (nasal septum) was perpendicular to the floor. Axially, the midsagittal plane was perpendicular to the floor (Figure 1). RR of the lateral incisors was graded using the categories described by Ericson and Kurol (12) (Table 1 foot note). The axes were adjusted on the maxillary lateral incisors in all planes to assess resorption. Axial and coronal views were used to determine the stage and level of lateral incisor RR. An example of a case of lateral incisor RR is shown in Figure 2. All CBCT images were also reviewed for incidental findings by the radiology department team.

**Table 1:** Root resorption of lateral incisors including the degree and location of the resorption.

Root resorption	*n* (%)
Root resorption categories	
No resorption (Grade 1)	112 (71.8)
Slight resorption (Grade 2)	28 (18.0)
Moderate resorption (Grade 3)	13 (8.3)
Severe resorption (Grade 4)	3 (1.9)
Location of root resorption	
Middle third of the root	21 (47.7)
Apical third of the root	23 (52.3)

Number of lateral incisors (*n*), percentage (%).

Grade 1: no resorption, intact root surface, with or without loss of cementum, Grade 2: Slight resorption up to half of the dentine thickness to the pulp. Grade 3: Moderate resorption, more than half way to the pulp. Grade 4: Severe resorption, exposed pulp. Ericson and Kurol (12).

### Clinical procedure and intervention (T0)

**Figure 3:**
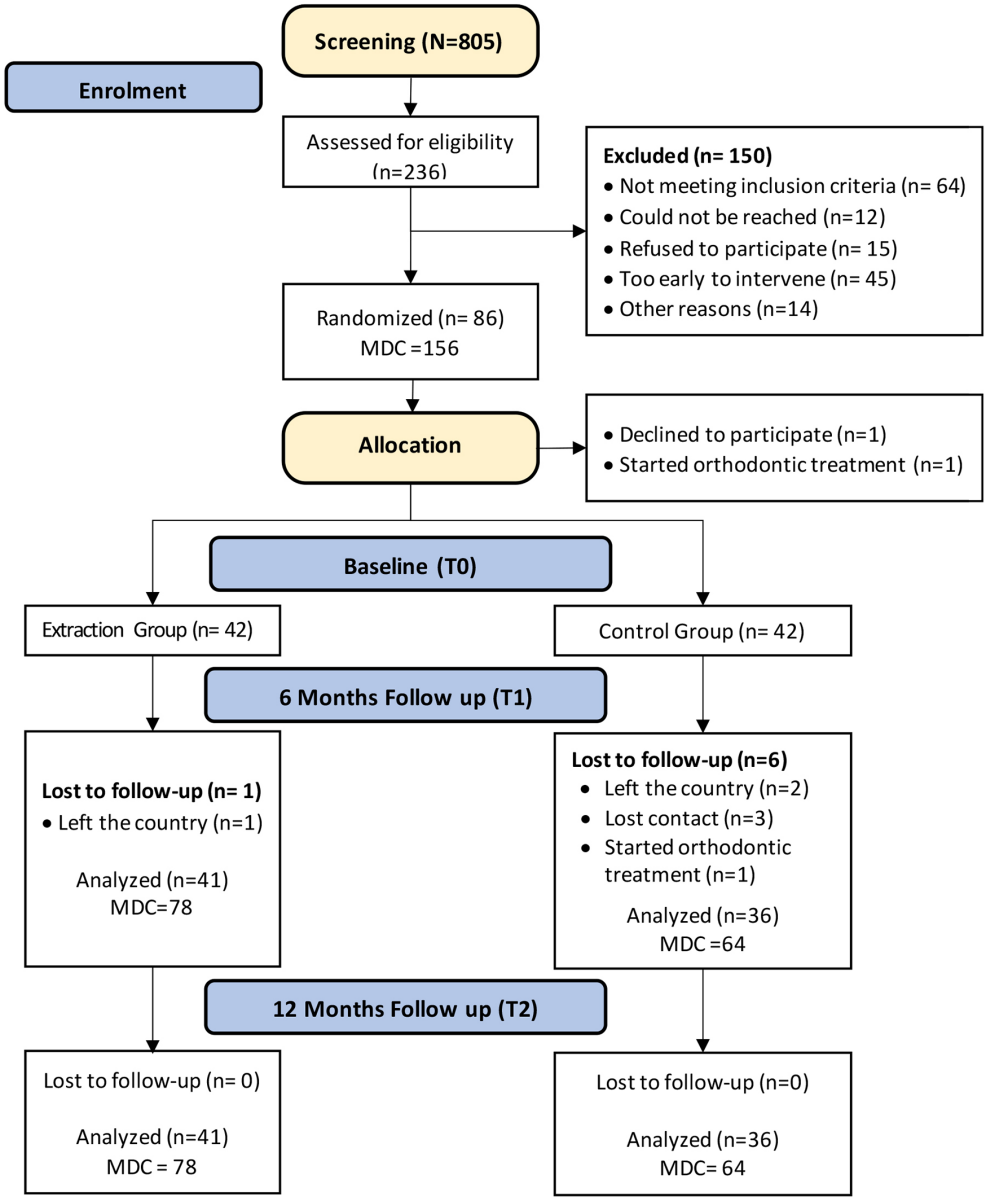
CONSORT flow diagram showing the flow of the patients included in the study up to 12-month follow-up period. *N*, total number of patients screened; *n*, number of patients in each subsample; MDC, mesioangular displaced canine.

On the day of baseline CBCT scanning (T0), extraction of the primary canines was performed by a single paediatric dentist. All patients in the EG had both primary canines extracted, regardless of whether the displacement was unilateral or bilateral. The flow of the patients in the study is summarised in Figure 3 and as follows:

### Follow-up (T1)

At the 6-month follow-up (T1), patients in both allocation groups underwent clinical examinations to check if their maxillary canines were positively palpated or clinically visible (partially or fully erupted). The conditions of primary canines were also noted; if they were extracted, exfoliated, firm, or mobile.

### Follow-up (T2)

At the 12-month follow-up (T2), the same clinical examination was repeated; for patients with clinically visible permanent canines, no further CBCTs were performed. For the other cases, a second CBCT scan was performed to compare the change in the MDC position with that shown in the baseline CBCT scan, resorption was re-assessed and progression was recorded. If children had canines that, according to clinical judgement, were expected to spontaneously erupt, these cases were followed up for a further 6 months until the canines were clinically visible. Periapical radiographs were taken for these cases to confirm the proximity of the canines to the gingival tissues.

Cases where the position of the canines had not improved radiographically and RR of the adjacent teeth had been recorded were placed on a high priority list that was given to the head of the orthodontic department.

### Statistical analyses

Data were analysed using SAS software (version 9.4; SAS Institute Inc., Cary, North Carolina, USA). Descriptive statistics are presented as frequencies and percentages. Cohen's kappa coefficient (*κ*) was used for intra-rater and inter-rater agreement of panoramic radiographic evaluation. The kappa values were classified as follows: <0, no agreement, 0–0.20 as slight, 0.21–0.40 as fair, 0.41–0.60 as moderate, 0.61–0.80 as substantial, and 0.81–1 as almost perfect agreement (28). The ICC was used to assess the intra-examiner reliability of the RR CBCT measurements. ICC values were classified as follows: <0.50, poor; 0.50–0.75, moderate; 0.75–0.90, good; and >0.90, excellent (29). Descriptive statistics were used to report data (mean, standard deviation, and percentages). Analysis of variance test was performed to test the association between the degree of angulation on panoramic radiographs and the degree of resorption on CBCT scans. To compare the angular and linear measurements in CBCT scans between the control and extraction groups at T0 and T2, a paired sample *t*-test was performed.

## Results

**Figure 4:**
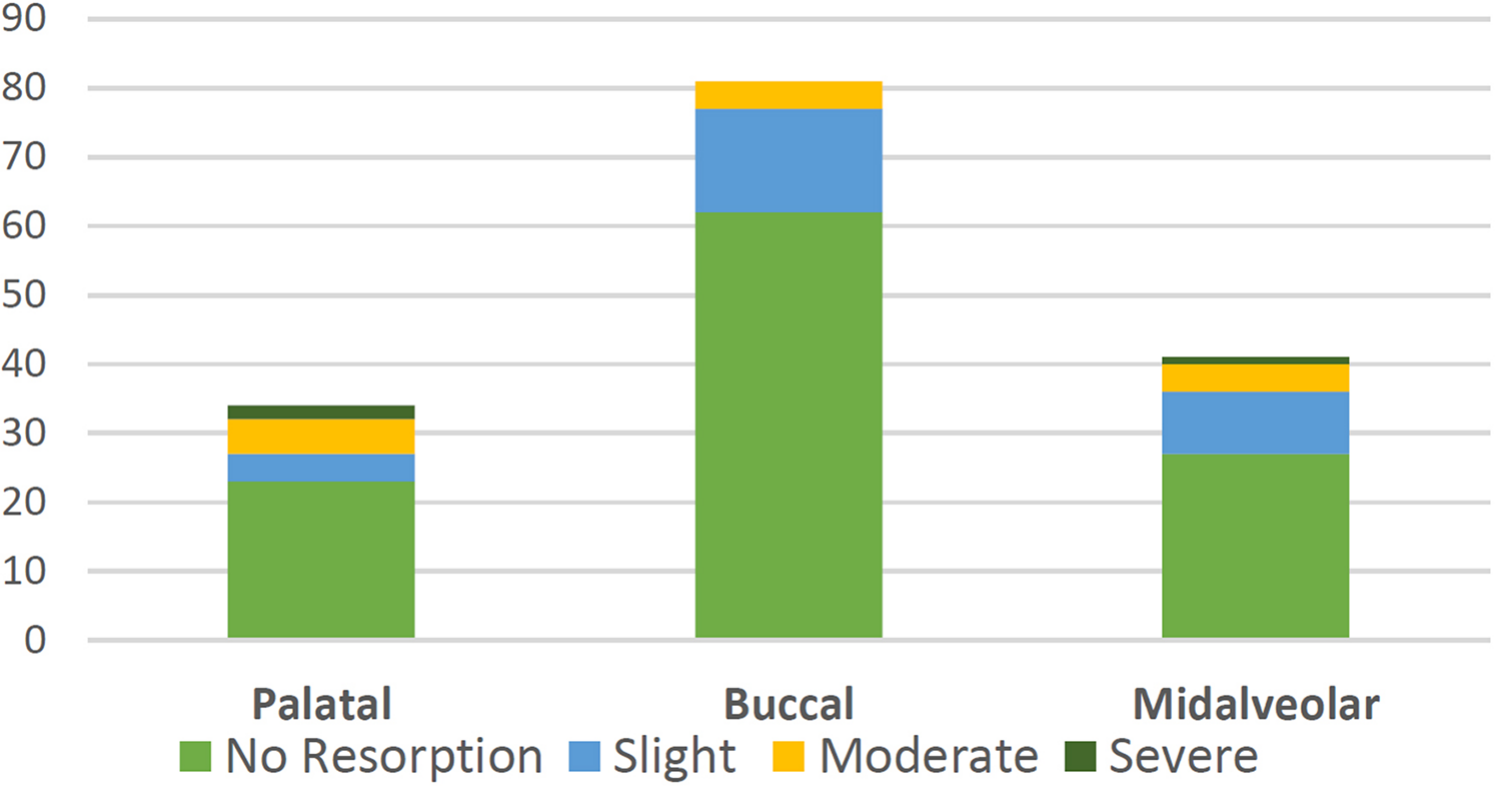
Degree of resorption in relation to the type of displacement.

**Figure 5:**
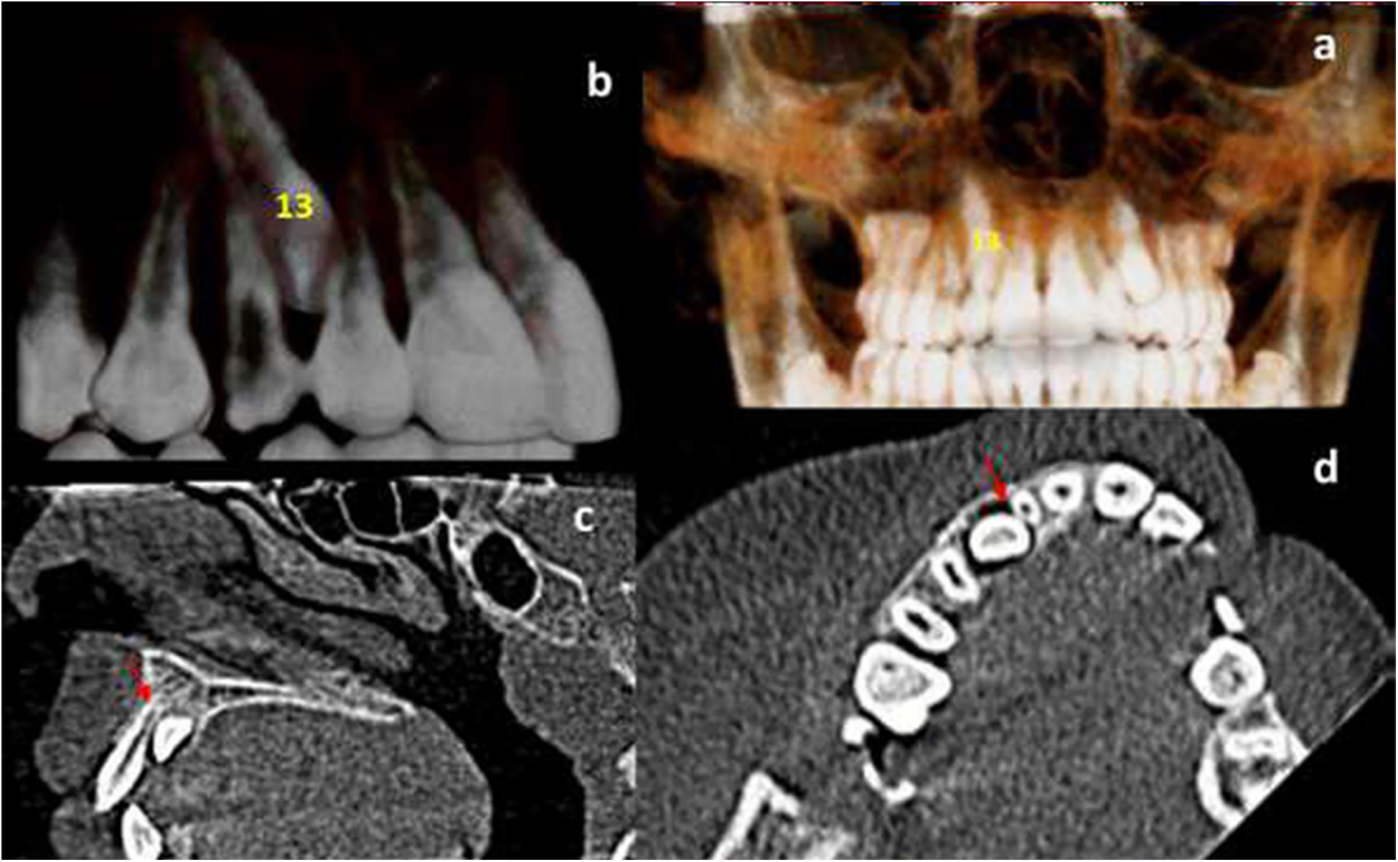
Cone-beam computed tomography (CBCT) view of a 12-year-old girl with unilateral right palatal canine displacement. **(a)** three-dimensional view showing the mesial inclination and palatal position of the right canine (tooth no. 13). **(b)** Close-up three-dimensional view of the displaced canine (13). **(c)** Sagittal view showing moderate degree of resorption of the right maxillary lateral incisor root “grade 3” (red arrow). **(d)** Axial view showing moderate degree of resorption of the right maxillary lateral incisor root (red arrow).

The prevalence of RR of the adjacent maxillary lateral incisors caused by displaced canines is summarised in (Table 1). RR was detected radiographically in 44 lateral incisors (28.2%). RR ranged from slight (18%) to severe resorption (1.9%). RR occurred in the apical third of the root in 52.3% of the cases and occurred in the middle third of the root in 47.7% of the cases. The minimum age at RR in this study was 9 years. RR occurred more frequently with midalveolar (34.1%) and palatal (32.3%) displacements than with buccal displacements (23.5%). More severe resorptions were more likely to occur with PDCs. However, the association between the type of displacement and the degree of resorption was not statistically significant (*P* = 0.14) (Table 2, Figure 4). There was no significant difference in the resorption level at T0 between the CG and the EG (*P* = 0.72) (Table 3). An example of a case with lateral incisor RR is shown in (Figure 5).

**Table 2:** Relation between the degree of resorption and type of displacement.

Type of displacement	Degree of resorption				Total number of teeth with root resorption *n* (%)	*P*-value
	No resorption (Grade 1) *n* (%)	Slight resorption (Grade 2) *n* (%)	Moderate resorption (Grade 3) *n* (%)	Severe resorption (Grade 4) *n* (%)		0.14 (NS)
Buccal (*N* = 81)	62 (76.5)	15 (18.5)	4 (4.9)	0	19 (23.5)	
Palatal (*N* = 34)	23 (67.7)	4 (11.8)	5 (14.7)	2 (5.9)	11 (32.3)	
Midalveolar (*N* = 41)	27 (65.9)	9 (22.0)	4 (9.8)	1 (2.4)	14 (34.1)	
Total Number	*n* = 112	*n* = 28	*n* = 13	*n* = 3	*n* = 44	

Fisher's Exact test, not-significant (NS), number of lateral incisors (*n*), total number of displaced canines in each group (*N*).

**Table 3:** Relation between the degree of lateral incisors' resorption and allocation group.

Root resorption	*n* (%)	Extraction *n* (%)	Control *n* (%)	*P*-value
Root resorption categories				0.72
No resorption (Grade 1)	112 (71.8)	54 (70.1)	58 (73.4)	
Yes (Grades 2–4)	44 (28.2)	23 (29.9)	21 (26.6)	
Root resorption categories				0.72
No resorption (Grade 1)	112 (71.8)	54 (70.1)	58 (73.4)	
Slight resorption (Grade 2)	28 (18.0)	13 (16.9)	15 (19.0)	
Moderate resorption (Grade 3)	13 (8.3)	8 (10.4)	5 (6.3)	
Severe resorption (Grade 4)	3 (1.9)	2 (2.6)	1 (1.3)	
Location of root resorption				0.99
Middle third of the root	21 (47.7)	11 (47.8)	10 (47.6)	
Apical third of the root	23 (52.3)	12 (52.2)	11 (52.4)	

Fisher's Exact test, total number of lateral incisors (*N*), number of lateral incisors in each allocation group (*n*).

The degree of resorption of the lateral incisors at baseline (T0) and 12-month follow-up (T2) were moderately positively associated. The Spearman rho correlation was 0.68 (*P* < 0.0001). The resorption significantly changed from baseline (T0) to 12-month follow-up (T2) (*P* = 0.0007) (Table 4). There was a significant increase in the degree of resorption between T2 and T0, with a mean difference equals to 0.31 (0.73), *P* < 0.0001 (Table 5). Using Student's *t*-test, there was no significant difference in the resorption level from T0 to T2 between the CG and EG (*P* = 0.11).

**Table 4:** The change of degree of resorption percentage between T0 and T2.

Degree of resorption *N* = 102	T0 *N* (%)	T2 *N* (%)	*P*-value
No resorption (Grade 1)	82 (80.4)	67 (65.7)	**0.0007** *
Slight resorption (Grade 2)	11 (10.8)	16 (15.7)	
Moderate resorption (Grade 3)	7 (6.9)	10 (9.8)	
Severe resorption (Grade 4)	2 (2.0)	9 (8.8)	
Spearman's rho (*r*)	0.68		**<0.0001** *

Total number of lateral incisors evaluated at baseline and 12-month follow-up (*N*). Spearman's correlation coefficient (*r*), baseline (T0), 12-month follow-up (T2).

The bold values represent “*Statistically significant”, Bowker's symmetry test.

**Table 5:** The difference of degree of resorption between T0 and T2.

T0 *N* = 102	T2 *N* = 102	Number of lateral incisors	*P*-value
No Change in the degree of resorption (*n* = 79)			
No resorption (Grade 1)		66	
Slight resorption (Grade 2)		4	
Moderate resorption (Grade 3)		7	
Severe resorption (Grade 4)		2	
Resorption Increased (*n* = 22)			
No resorption (Grade 1)	Slight resorption (Grade 2)	12	
No resorption (Grade 1)	Severe resorption (Grade 4)	4	
Slight resorption (Grade 2)	Moderate resorption (Grade 3)	7	
Slight resorption (Grade 2)	Severe resorption (Grade 4)	2	
Mean difference between T0 and T2			
**Mean (SD)**			
0.31 (0.73)			**<0.0001** *

Total number of lateral incisors in each subsample (*n*). Standard deviation (SD), baseline (T0), 12-month follow-up (T2).

The bold values represent “*Statistically significant”, paired *t*-test. Total number of lateral incisors evaluated at baseline and 12-month follow-up (*N*).

## Discussion

The aim of this randomised clinical trial was to assess the effect of interceptive extraction of the primary canines on the condition of the roots of permanent neighbouring teeth to MDCs. In addition, we evaluated the association between RR and a certain type of displacement. The results showed no significant differences in the interceptive extraction of the primary canines between the control and study groups. Moreover, there was no significant difference between the type of displacement and degree of resorption.

Since panoramic radiography is considered among the indispensable tools used for diagnosis by orthodontists. However, panoramic radiographs were short in estimating the degree of actual RR, and overestimated the angle of inclination by 13° (30, 31). This justify the need for CBCT examinations for the proper evaluation of RR, and reveal that the accuracy of 3D radiographic techniques (CBCT) is of critical importance in determining the exact canine position in contrast to conventional panoramic radiographs. CBCT images considerably improved the ability to detect and quantify RR. It is important to consider the extent to which canine displacement is associated with RR of the adjacent teeth when planning treatment. At baseline in our study, RR was detected in 28.2% of adjacent maxillary lateral incisors: 18% had slight resorption, 8.3% had moderate resorption, and 1.9% had severe resorption. Comparable results were found by Liu et al. and Ericson and Kurol, who detected resorption in 27.2% and 38% of lateral incisors, respectively (3, 12). In contrast, the RR identified in the current study was higher than that reported by Ericson and Kurol in 1988 which was only 12.5% (25). However, our percentage was lower than those reported by Walker et al. and Kim et al. which were 66.7% and 49.5%, respectively (16, 30). These differences could have been caused by sampling, age distribution, radiography technique (2D vs. 3D), and type of scanner (CT vs. CBCT) (10, 12) or not discussing the scanning parameters used for CBCT imaging, namely the voxel size, scanning time, and FOV (3).

The occurrence of severe resorption in adjacent lateral incisors in our study was low (1.9%) compared with that in other studies, which reported occurrences of (22.8%) (5), (12.6%) (32), and (5.2%) (3). In addition, no resorption was detected in the adjacent central incisors or premolars because all cases of horizontal displacement and severe degrees of displacement were excluded.

RR was detected in patients as young as 9 years old. Similar results were reported by Ericson and Kurol, who found severe resorption in 9-year-old patients (5). This highlights the importance of early evaluation of canines' path of eruption to avoid the occurrence of complications at this early stage. RR occurred more frequently in patients with palatal and midalveolar displacements than in those with buccal displacements. This is in agreement with the finding reported by Ericson and Kurol, who detected more resorption when canines were positioned palatally or distopalatally (10). However, the relationship between the type of displacement and RR in our study did not reach statistical significance. In contrast, other studies have shown that severe resorption occurs more frequently in patients with buccal canine displacement (30, 32).

Different parts of the roots may be affected by resorption. In our study, RR occurred in 52.3% of the apical one-third. This was similarly presented in a recent systematic review, which stated that the most frequent position of RR was at the apical one-third (56.87%) (4). Similarly, Crenochova et al. found RR to be located in the apical third in 57.6% of cases (32). In contrast, an older study by Ericson and Kurol in 1987 found that most resorptions (82%) occurred in the middle third of the root (10). However, a more recent study by Ercison and Kurol, in which they used CT, found that 43% of resorptions occurred in the apical third (12).

At the 12-month follow-up, there was a significant increase in the degree of resorption between T2 and T0, and the change in the resorption level from T0 to T2 was similar between the control and extraction groups. The literature concerning root resorption associated with early orthodontic intervention remains scarce. The majority of available literature investigating root resorption (RR) has centered on the impact of untreated impacted canines. This study addresses a significant gap in the literature by examining root resorption in the context of early orthodontic treatment. A single study reported a low incidence of root resorption affecting both central and lateral incisors on the same side due to impacted canines in the absence of treatment (33). This might be an opposite to what a recent systematic review suggested that RR of lateral incisors, while reported at a relatively high incidence of 50% (34), compared to 28.2% in the present investigation. This discrepancy might be partially explained by variations in diagnostic criteria employed across studies, including the definition of resorption severity and the imaging modalities used. Notably, a substantial heterogeneity in RR incidence was evident across included studies, hindering definitive conclusions. The influence of canine angulation on resorption severity remains unclear due to inconsistent data reporting (34). To observe a discernible difference in root resorption, a more extended follow-up period without treatment might be necessary. However, this presents an ethical conundrum as most patients were awaiting orthodontic intervention. Which often involves, early extraction of primary canines as it has been recommended (35). In this present investigation, the degree of resorption progressed to severe resorption in three cases at follow-up, and the other three cases progressed to moderate resorption. Twelve new cases showed signs of slight resorption, and four new cases showed signs of severe resorption. Our study is one of few that followed up with patients presenting with resorbed teeth and reassessed their lateral incisors for signs of new resorptive lesions. A recent systematic review commented on the lack of studies that followed up patients with resorbed teeth (4). This highlights the importance of following up the cases with lateral incisor RR associated with canine displacements and re-asses them. Caution should be taken when evaluating resorption progression; in seven teeth of this study, the rate of progression was high. This also highlights the importance of quick referrals to orthodontic departments in canine displacement cases, particularly when resorption is present, and collaboration between the departments in such cases is crucial. Keeping in mind that the orthodontic force applied to the tooth is transmitted through the periodontal ligament to the alveolar bone, triggering bone resorption and apposition. However, the exact mechanism by which this force initiates bone remodeling remains unclear, with the periodontal ligament playing a crucial role as a soft tissue interface (36). This could lead to both desired and not desired outcome. Further research is needed to fully understand these complex interactions and develop more effective orthodontic treatment strategies. Further important clinical implication should emphasise on understanding the potential for RR in lateral incisors associated with displaced canines is essential for patient counselling. Pediatric dentists and orthodontists can inform patients about the risk of RR and the importance of regular check-ups to monitor the condition. While the study provides valuable insights, further research is needed to investigate the factors influencing the severity and progression of RR, as well as to develop effective preventive or therapeutic strategies once diagnosed. Additionally, investigating further the role of dental follicle volume and periodontal ligament or any other factors that might affect this phenomena (17, 19, 36).

### Limitations of the study

This study was also one of the few studies to not only study the degree of RR in adjacent lateral incisors but also the rate of progression of RR for a total of 12 months. To ethically limit cumulative radiation exposure, we refrained from performing a CBCT scan at the 6-month follow-up (T1), opting instead for the 12-month follow-up (T2) to assess root resorption progression. This decision, though it introduced a gap in mid-term data, was necessary to prioritize patient safety. Additionally, we selected a lower resolution (0.4 voxels) to minimize radiation, despite the potential technical limitations this introduced, such as partial volume averaging artifacts. The choice of a larger field of view (16 × 6 cm) was also guided by the available CBCT machine capabilities, as using a smaller FOV would have necessitated a higher radiation dose, which we aimed to avoid. While these adjustments may limit the study's precision, they were essential to protect the pediatric patients involved.

The limitation associated with CBCT images was related to the selected resolution, which was 0.4 voxels. We noticed that few cases were diagnosed with lateral incisor resorption at baseline scan; however, we were not able to detect resorption in the follow-up scan at the same location (middle or apical third of the root) after 1 year of follow-up. These cases were considered false positives and were excluded. This could be related to technical errors in the images owing to the low-resolution settings. A systematic error that might have occurred is the partial volume averaging, which occurs when the voxel size is larger than the object to be imaged (37). This occurs more often along the boundaries of objects (38). When this artifact occurs, thin objects can appear thinner than they truly are, and they can even disappear on CBCT scans (37). On the other hand, cases with resorption at the same location on both scans (baseline and 12-month follow-up) were more likely to have true resorption and the chance of this being explained by a technical error was much less. The most effective way to reduce this error is to use a smaller voxel size (higher resolution) (37). Low resolution was chosen in this study because the age group of our sample was between 9 and 12 years, who were more prone to radiation risks. In addition, the available CBCT machine in our hospital had a FOV of 16 × 6 as the smallest, so we were not able to improve the resolution by selecting an FOV smaller than 16 × 6 in order to keep the radiation dose as low as possible. Nevertheless, a higher resolution with smaller FOV if possible is highly recommended for future studies.

## Conclusion

• Interceptive extraction of primary canines did not significantly influence the occurrence or severity of root resorption (RR) in adjacent lateral incisors.
• RR was detected in 28.2% of adjacent lateral incisors to MDC at baseline.
• RR increased significantly between baseline and 12-month follow-up.
• CBCT imaging is recommended for accurate diagnosis and treatment planning, particularly, in cases involving MDC where clinical or panoramic findings indicate a high risk of root resorption for adjacent teeth.

Within the limitations of this study, root resorption is a common complication in patients with displaced canines, and it progress over time. Interceptive extraction of primary canines did not impact the occurrence or severity of root resorption in adjacent lateral incisors. However, careful monitoring is essential for early detection and intervention. CBCT imaging is recommended for accurate diagnosis and treatment planning where clinical or panoramic findings indicate a high risk of root resorption. Further research is needed to investigate other treatment modalities that might have an effect on RR.

## Data Availability

The original contributions presented in the study are included in the article/Supplementary Material, further inquiries can be directed to the corresponding author.

## References

[1] Alqerban A Jacobs R Lambrechts P Loozen G Willems G. Root resorption of the maxillary lateral incisor caused by impacted canine: a literature review. Clin Oral Investig. (2009) 13(3):247–55. 10.1007/s00784-009-0262-8 19277728

[2] Husain J Burden D McSherry P Morris D Allen M, Clinical Standards Committee of the Faculty of Dental Surgery, Royal College of Surgeons of England. National clinical guidelines for management of the palatally ectopic maxillary canine. Br Dent J. (2012) 213(4):171–6. 10.1038/sj.bdj.2012.726 22918345

[3] Liu D Zhang W Zhang Z Wu Y Ma X. Localization of impacted maxillary canines and observation of adjacent incisor resorption with cone-beam computed tomography. Oral Surg Oral Med Oral Pathol Oral Radiol Endod. (2008) 105(1):91–8. 10.1016/j.tripleo.2007.01.030 17507268

[4] Schroder AGD Guariza-Filho O de Araujo CM Ruellas AC Tanaka OM Porporatti AL. To what extent are impacted canines associated with root resorption of the adjacent tooth?: a systematic review with meta-analysis. J Am Dent Assoc. (2018) 149(9):765–77.e8. 10.1016/j.adaj.2018.05.012 30165975

[5] Ericson S Kurol PJ. Resorption of incisors after ectopic eruption of maxillary canines: a CT study. Angle Orthod. (2000) 70(6):415–23. 10.1043/0003-3219(2000)070<0415:ROIAEE>2.0.CO;2 11138644

[6] Jacoby H. The etiology of maxillary canine impactions. Am J Orthod. (1983) 84(2):125–32. 10.1016/0002-9416(83)90176-8 6576636

[7] Kapila S Conley RS Harrell WE. The current status of cone beam computed tomography imaging in orthodontics. Dentomaxillofac Radiol. (2011) 40(1):24–34. 10.1259/dmfr/12615645 21159912 PMC3611465

[8] Botticelli S Verna C Cattaneo PM Heidmann J Melsen B. Two- versus three-dimensional imaging in subjects with unerupted maxillary canines. Eur J Orthod. (2011) 33(4):344–9. 10.1093/ejo/cjq102 21131389

[9] Haney E Gansky SA Lee JS Johnson E Maki K Miller AJ Comparative analysis of traditional radiographs and cone-beam computed tomography volumetric images in the diagnosis and treatment planning of maxillary impacted canines. Am J Orthod Dentofacial Orthop. (2010) 137(5):590–7. 10.1016/j.ajodo.2008.06.035 20451777

[10] Ericson S Kurol J. Incisor resorption caused by maxillary cuspids. A radiographic study. Angle Orthod. (1987) 57(4):332–46. 10.1043/0003-3219(1987)057<0332:IRCBMC>2.0.CO;2 3479035

[11] Alqerban A Hedesiu M Baciut M Nackaerts O Jacobs R Fieuws S Pre-surgical treatment planning of maxillary canine impactions using panoramic vs cone beam CT imaging. Dentomaxillofac Radiol. (2013) 42(9):20130157. 10.1259/dmfr.20130157 23906975 PMC3828021

[12] Ericson S Kurol J. Incisor root resorptions due to ectopic maxillary canines imaged by computerized tomography: a comparative study in extracted teeth. Angle Orthod. (2000) 70(4):276–83. 10.1043/0003-3219(2000)070<0276:IRRDTE>2.0.CO;2 10961776

[13] Bjerklin K Ericson S. How a computerized tomography examination changed the treatment plans of 80 children with retained and ectopically positioned maxillary canines. Angle Orthod. (2006) 76(1):43–51. 10.1043/0003-3219(2006)076[0043:HACTEC]2.0.CO;2 16448268

[14] Oenning AC Jacobs R Pauwels R Stratis A Hedesiu M Cone-beam CT in paediatric dentistry: DIMITRA project position statement. Pediatr Radiol. (2018) 48(3):308–16. 10.1007/s00247-017-4012-9 29143199

[15] Littlewood SJ Mitchell L. An Introduction to Orthodontics. 5th edn. Oxford, UK: Oxford University Press (2019).

[16] Walker L Enciso R Mah J. Three-dimensional localization of maxillary canines with cone-beam computed tomography. Am J Orthod Dentofacial Orthop. (2005) 128(4):418–23. 10.1016/j.ajodo.2004.04.033 16214621

[17] Kjær I. Mechanism of human tooth eruption: review article including a new theory for future studies on the eruption process. Scientifica (Cairo). (2014) 2014:341905. 10.1155/2014/341905 24688798 PMC3944225

[18] Ericson S Bjerklin K. The dental follicle in normally and ectopically erupting maxillary canines: a computed tomography study. Angle Orthod. (2001) 71(5):333–42. 10.1043/0003-3219(2001)071<0333:TDFINA>2.0.CO;2 11605866

[19] Lam M Dekel E Nucci L Grassia V Naoumova J Pacheco-Pereira C The effect of the dental follicle volume of palatally impacted canines on the relative position of the adjacent teeth. Eur J Orthod. (2024) 46(1):cjad071. 10.1093/ejo/cjad071 38001047

[20] Heboyan A Avetisyan A Karobari MI Marya A Khurshid Z Rokaya D Tooth root resorption: a review. Sci Prog. (2022) 105(3):368504221109217. 10.1177/00368504221109217 35759366 PMC10358711

[21] Nolla CM. The development of the permanent teeth. J Dent Child. (1960) 27:254–66.

[22] Naoumova J Kurol J Kjellberg H. Extraction of the deciduous canine as an interceptive treatment in children with palatal displaced canines—part I: shall we extract the deciduous canine or not? Eur J Orthod. (2015) 37(2):209–18. 10.1093/ejo/cju040 25246604

[23] Naoumova J Kürol J Kjellberg H. Extraction of the deciduous canine as an interceptive treatment in children with palatally displaced canines—part II: possible predictors of success and cut-off points for a spontaneous eruption. Eur J Orthod. (2015) 37(2):219–29. 10.1093/ejo/cju102 25700993

[24] Moher D Hopewell S Schulz KF Montori V Gøtzsche PC Devereaux P CONSORT 2010 explanation and elaboration: updated guidelines for reporting parallel group randomised trials. Int J Surg. (2012) 10(1):28–55. 10.1016/j.ijsu.2011.10.001 22036893

[25] Ericson S Kurol J. Resorption of maxillary lateral incisors caused by ectopic eruption of the canines. A clinical and radiographic analysis of predisposing factors. Am J Orthod Dentofacial Orthop. (1988) 94(6):503–13. 10.1016/0889-5406(88)90008-x 3195514

[26] Stivaros N Mandall NA. Radiographic factors affecting the management of impacted upper permanent canines. J Orthod. (2000) 27(2):169–73. 10.1093/ortho/27.2.169 10867073

[27] Rosenberger W Lachin J. Randomization in Clinical Trials: Theory and Practice. Hoboken, NJ: John Wiley & Sons Inc. (2002). 10.1002/0471722103

[28] Landis JR Koch GG. The measurement of observer agreement for categorical data. Biometrics. (1977) 33(1):159–74. 10.2307/2529310 843571

[29] Koo TK Li MY. A guideline of selecting and reporting intraclass correlation coefficients for reliability research. J Chiropr Med. (2016) 15(2):155–63. 10.1016/j.jcm.2016.02.012 27330520 PMC4913118

[30] Kim Y Hyun H-K Jang K-T. The position of maxillary canine impactions and the influenced factors to adjacent root resorption in the Korean population. Eur J Orthod. (2012) 34(3):302–6. 10.1093/ejo/cjr002 21303809

[31] Dalessandri D Migliorati M Visconti L Contardo L Kau CH Martin C. KPG index versus OPG measurements: a comparison between 3D and 2D methods in predicting treatment duration and difficulty level for patients with impacted maxillary canines. Biomed Res Int. (2014) 2014:537620. 10.1155/2014/537620 25126566 PMC4119896

[32] Cernochova P Krupa P Izakovicova-Holla L. Root resorption associated with ectopically erupting maxillary permanent canines: a computed tomography study. Eur J Orthod. (2011) 33(5):483–91. 10.1093/ejo/cjq085 21127168

[33] Kalavritinos M Benetou V Bitsanis E Sanoudos M Alexiou K Tsiklakis K Incidence of incisor root resorption associated with the position of the impacted maxillary canines: a cone-beam computed tomographic study. Am J Orthod Dentofacial Orthop. (2020) 157(1):73–9. 10.1016/j.ajodo.2019.02.016 31901285

[34] Mitsea A Palikaraki G Karamesinis K Vastardis H Gizani S Sifakakis I. Evaluation of lateral incisor resorption caused by impacted maxillary canines based on CBCT: a systematic review and meta-analysis. Children (Basel). (2022) 9(7):1006. 10.3390/children9071006 35883990 PMC9323464

[35] Almasoud NN. Extraction of primary canines for interceptive orthodontic treatment of palatally displaced permanent canines: a systematic review. Angle Orthod. (2017) 87(6):878–85. 10.2319/021417-105.1 28800259 PMC8317556

[36] Tepedino M. The mechanical role of the periodontal ligament for developing mathematical models in orthodontics. Math Mech Complex Syst. (2023) 11:525–39. 10.2140/memocs.2023.11.525

[37] Molen AD. Considerations in the use of cone-beam computed tomography for buccal bone measurements. Am J Orthod Dentofacial Orthop. (2010) 137(4 Suppl):S130–135. 10.1016/j.ajodo.2010.01.015 20381753

[38] Scarfe WC Farman AG. What is cone-beam CT and how does it work? Dent Clin North Am. (2008) 52(4):707–30 v. 10.1016/j.cden.2008.05.005 18805225

